# Brain areas modulation in consciousness during sevoflurane anesthesia

**DOI:** 10.3389/fnint.2022.1031613

**Published:** 2022-12-21

**Authors:** Jie Lyu, Huajing Cai, Yeru Chen, Gang Chen

**Affiliations:** Department of Anesthesiology, Sir Run Run Shaw Hospital, School of Medicine, Zhejiang University, Hangzhou, Zhejiang, China

**Keywords:** sevoflurane, general anesthesia, consciousness, circuits, loss of righting reflex, recovery of righting reflex

## Abstract

Sevoflurane is presently one of the most used inhaled anesthetics worldwide. However, the mechanisms through which sevoflurane acts and the areas of the brain associated with changes in consciousness during anesthesia remain important and complex research questions. Sevoflurane is generally regarded as a volatile anesthetic that blindly targets neuronal (and sometimes astrocyte) GABAA receptors. This review focuses on the brain areas of sevoflurane action and their relation to changes in consciousness during anesthesia. We cover 20 years of history, from the bench to the bedside, and include perspectives on functional magnetic resonance, electroencephalogram, and pharmacological experiments. We review the interactions and neurotransmitters involved in brain circuits during sevoflurane anesthesia, improving the effectiveness and accuracy of sevoflurane’s future application and shedding light on the mechanisms behind human consciousness.

## 1. Introduction

Sevoflurane has been one of the most widely used inhaled anesthetics in a variety of surgery interventions over the past 30 years (O’Keeffe and Healy, 1999). This drug presents several advantages, including quick onset, fewer side effects, less irritation, and lower blood gas ratio compared to other inhaled anesthetics (Ghatge et al., 2003). Despite its importance in modern volatile anesthesia, the precise mechanism through which sevoflurane induces general anesthesia, and which parts of the brain are associated to induce loss of consciousness remain unknown.

### 1.1 Previously mentioned pathway of sevoflurane anesthesia

**Figure 1:**
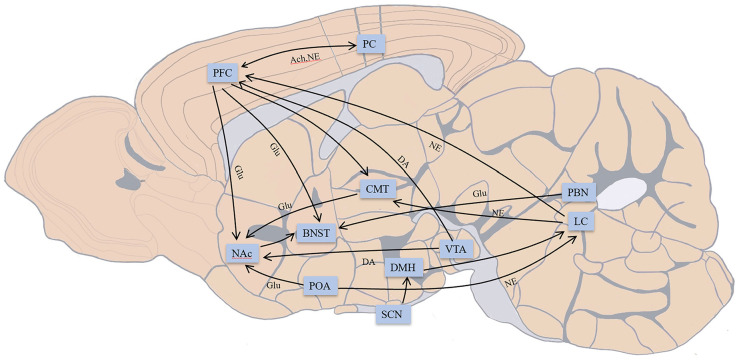
Main connections in brain areas related to conscious changes during sevoflurane anesthesia; not all connections are shown. The connection between the prefrontal cortex (PFC) and parietal cortex (PC) participates in fronto-parietal network. PFC provides descending projections to bed nucleus of stria terminalis (BNST), nucleus accumbens (NAc), centromedial nucleus of the thalamus (CMT), and receives inputs from the ventral tegmental area (VTA), locus coeruleus (LC). CMT receives input from LC, and PFC and outputs to NAc. The preoptic area (POA) connects with LC and projects to NAc. Indirect projections from the suprachiasmatic nucleus (SCN)-dorsomedial hypothalamic nucleus (DMH)-LC function as an arousal system in the brain. VTA provides dopaminergic projections to NAc. LC outputs noradrenergic projections over the whole brain. BNST receives glutamatergic input from the parabrachial nucleus (PBN). NAc projects to BNST may participate in the consciousness changes during sevoflurane anesthesia. Excitatory neurotransmitters: Glu, glutamate; DA, dopamine; NE, norepinephrine; Ach, acetylcholine. Inhibitory neurotransmitters: GABA, γ-aminobutyric acid.

Some previous reviews covered the influence of drugs or the pathological caused inactivation of different brain areas exposed to general anesthetics based on pharmacological experiments and the function of these brain regions and explored the relationship between conventional sleep-wake structures and anesthetic action pathways (Franks, 2008; Brown et al., 2010; Leung et al., 2014). According to previous research, sevoflurane acts on GABA receptors throughout the brain in a similar manner to inhalation anesthetics (Hemmings et al., 2019). Such anesthetics can affect the pons, midbrain, and hypothalamus, inhibit the ascending reticular activating system, diminish the output of the arousal pathway to the cortex, and finally cause atonia (Brown et al., 2011). Previous evaluations mostly ignored the differences in brain regulation of various anesthetics and instead concentrated on the similar action pathways of anesthetic medicines, such as the fronto-parietal circuit and thalamus (Hudetz, 2012). Only a tiny part of the connections between brain regions in the previous reviews is certainly related to the conscious changes induced by sevoflurane. The current review will concentrate on the linkages between the brain’s regions that have been linked to sevoflurane anesthesia throughout the previous 20 years. Some recently identified circuits, including the midbrain dopaminergic pathway, significant arousal nuclei in the thalamus, hypothalamus, and pons, will also be discussed on their involvement in sevoflurane in addition to classical pathways including fronto-parietal and thalamocortical circuits. We begin by showing the brain connections associated with sevoflurane anesthesia in the schematic diagram (Figure 1).

### 1.2 Tools in sevoflurane anesthesia research

Sevoflurane anesthesia has a close correlation with the state of consciousness accompanied by loss of consciousness (LOC) and recovery of consciousness (ROC). In humans, consciousness is manifested by processing and integrating external information (Tononi, 2004), whereby anesthesia results not only in a lack of response to the environmental stimuli but also in the uncoupling between external information and individuals (Sanders et al., 2012). Hence, a universally used measure of LOC is the lack of movement in human subjects.

EEG is commonly used to evaluate the state of consciousness under anesthesia more accurately in many surgical contexts, as well as in experimental settings measuring the activity of brain areas in model animal organisms during sevoflurane anesthesia. Typically, this is achieved through a series of monitoring indicators derived from EEG, such as bispectral index, 95% spectral edge frequency, median frequency, and entropy index (Silva et al., 2018). In addition, the righting reflex (RR) is the most commonly used behavioral indicator in sevoflurane experimental settings to assess animal consciousness, with the loss of the righting reflex (LORR) and the recovery of the righting reflex (RORR) representing LOC and ROC, respectively (Gao and Calderon, 2020). The time-consuming and minimum alveolar concentration (MAC) of LORR or RORR is the most common way to illustrate the sensitivity of the experimental animals in sevoflurane studies.

By precisely modulating the neurons in particular brain regions, optogenetics, chemogenetics, and electrical stimulation can aid researchers in better understanding the function of brain regions in regulating consciousness while investigating brain regions related to anesthesia. These techniques can target a particular type of neuron or glial cell and investigate how it regulates anesthesia (Melonakos et al., 2020).

Sevoflurane can be a useful tool for researching the brain regions associated with consciousness since it is so closely linked to changes in consciousness. This can assist make the application of sevoflurane during surgeries more precise and tailored. The therapeutic use of sevoflurane and upcoming research on the mechanisms of anesthesia and awareness will benefit greatly from a review that compiles the most recent studies on the potential modification of brain areas during sevoflurane anesthesia.

## 2. Brain Areas and Circuits

### 2.1 Thalamocortical circuit

The thalamocortical circuit is vital for the regulation of LOC and ROC during sevoflurane anesthesia. The increase of K^+^ current, partial cortical deafferentation, and broken balance between excitation and inhibition may lead to instability of the thalamocortical network, resulting in unconsciousness (Massimini et al., 2012). The use of electrodes to stimulate the ventrobasal nucleus of the mice’s thalamus leads to the depolarization of the cortex. In contrast, the application of sevoflurane significantly decreased the intensity and degree of cortical depolarization, and prolonged thalamocortical signal transmission induced by stimulating the ventrobasal nucleus of the mice thalamus in a dose-dependent manner (Kratzer et al., 2017). Moreover, recording the EEG of the prefrontal cortex (PFC) and central thalamus (CT) under low-dose sevoflurane revealed amplification of intracranial local field potentials of PFC-CT and intracranial beta/low gamma (12–40 Hz) coherence of PFC-PFC. Slow delta oscillations (0.1–4 Hz) gradually became dominant in response to a higher dose of sevoflurane to induce LORR, with significant decreases in PFC-CT beta/low gamma coherence and EEG activity (Guidera et al., 2017). Instead of a holistic function pattern, different components of the thalamocortical circuit may take on a distinctive role in sevoflurane anesthesia (Velly et al., 2007). For example, during maintained sevoflurane anesthesia, EEG values decreased dramatically at LOC but electrography thalamus values remained unchanged. Moreover, electrography of the thalamus more precisely predicts motor response movements. Different electrography assays of the cortex and thalamus in response to sevoflurane administration suggest that changes in consciousness during sevoflurane anesthesia are mainly connected to the cerebral cortex, while thalamic anesthetic agents regulate the inhibition of movement response to nociceptive stimulus. However, it should be noted that subcortical electrogenesis may be defective in measuring the activity of deep brain structures while being efficient for detecting cortex activity (Jäntti et al., 2008), due to the use of only two electrodes. Hence, measuring cortical and subcortical structural functions during sevoflurane anesthesia requires more precise electrosubcorticograms and experimental inactivation of relevant brain regions.

#### 2.1.1 Neocortex

Increased extracellular concentration of dopamine was found in rat cortical slices after sevoflurane application, independent of the intracellular and extracellular Ca^2+^ concentration, demonstrating cortical effects of sevoflurane (Silva et al., 2007). Neocortical neurons show high-frequency action potential firing and episodes of low discharge activity among the connections between different regions of the neocortex, which has been linked to the state of consciousness during sevoflurane anesthesia (Drexler et al., 2013). The connectivity and integration across the frontal and parietal cortices have a crucial relationship to both LOC and ROC, whereby the connectivity between these two brain regions is regarded as the foundation for conscious changes during anesthesia (Alkire et al., 2008), including sevoflurane-induced unconsciousness. Comparisons of functional magnetic resonance images between patients anesthetized with sevoflurane and those presenting with brain injury showed that a discrepancy between the sensory and motor areas, their overall similarity, and dynamic change were closely associated with complete consciousness linked to the activity of the fronto-parietal network (Golkowski et al., 2021). In fact, the whole prefrontal cortex seems to have a significant correlation with consciousness, including the dorsomedial, dorsolateral and ventromedial prefrontal cortices, as measured by fisher scores by using vector machines. At the same time, sevoflurane anesthesia in humans has been linked to reduced connectivity between the middle frontal gyrus, the right fronto-parietal network, and the intraparietal regions of the brain (Golkowski et al., 2019). Functional magnetic resonance imaging had shown similar results in a change of consciousness studies during sevoflurane anesthesia in monkeys (Uhrig et al., 2018). While awake, a network of positive correlations is formed between the anterior and posterior cingulate cortices, the dorsomedial prefrontal cortex, and other visual, auditory, and motor areas of the brain. After sevoflurane anesthesia, the average coupling in the above brain regions decreased. More precisely, sevoflurane significantly isolated visual field areas and premotor areas in the frontal cortex from frontal-cingulate and fronto-parietal connections. However, the drug did not simply produce inhibition or isolation effects but also preserved (and even enhanced) functional connectivity within the frontal cortex. When brain structural rearrangement occurs, some local connections are expendable in order to preserve the main connections and brain function. In fact, the hierarchical disruption of the cortex is a feature of sevoflurane anesthesia, as shown by intrinsic ignition analysis. The affected areas include the cingulate cortex, the parietal cortex, the right frontal eye field, the left parahippocampal cortex, and the left primary somatosensory cortex (Signorelli et al., 2021). These results indicate that the entire fronto-parietal-cingulate network participates in the regulation of changes in consciousness changes during sevoflurane anesthesia.

Frequency alterations on the EEG also showed effects on cortical areas regulating the state of consciousness during sevoflurane anesthesia, with increased delta, theta, and alpha, and decreased beta and gamma. After analyzing the normalized symbol transfer entropy of the fronto-parietal network, the EEG showed that the decreasing feedback dominance and asymmetrical feedback/feedforward of the fronto-parietal network in baseline conscious and unconscious states were involved in the neural correlation of sevoflurane-induced unconsciousness. This also demonstrated that sevoflurane anesthesia caused disruption of the fronto-parietal network connectivity (Lee et al., 2013). The differences in the fronto-parietal network during sevoflurane anesthesia occur in both adults and infants, with the delta oscillation being predominant in the latter during both the maintenance and emergence periods. Global modularity increases and complexity decreases during the maintenance period of sevoflurane anesthesia as compared to the emergence period, with a significant difference in delta oscillatory connectivity of the fronto-parietal network (Pappas et al., 2019). In rats, both frontal and parietal EEG showed a coherent slow delta (0.1–4 Hz) during sevoflurane-induced unconsciousness. The increase of coupling appeared on delta-low gamma and theta-low gamma, restoring consciousness to the baseline of awake levels during the recovery period after sevoflurane anesthesia (Pal et al., 2017). Furthermore, low doses of sevoflurane increase the power of beta-low gamma in frontal and parietal EEG in the first minutes of LOC (Guidera et al., 2017). These EEG results indicate the involvement of the fronto-parietal network in sevoflurane-induced paradoxical excitation.

However, further studies on interlobar connectivity challenge the idea that drug-mediated unconsciousness reflects the functional integration of the cerebral cortex (Pal et al., 2020). On the one hand, the observed changes may be simultaneously caused by different conscious states and drug use. On the other hand, the limitation occurs when cortical connectivity, dynamics, and oscillations are adopted to estimate the level of consciousness. Cholinergic stimulation of the prefrontal cortex restores consciousness and induces awakening-like movements in rats, but does not alter the consciousness state in parietal regions (Pal et al., 2018). A growing amount of evidence demonstrates there are differences between the frontal and parietal regions in the modulation of consciousness changes induced by sevoflurane. For example, gamma connectivity of the prefrontal cortex did not recover after the emergence period following sevoflurane anesthesia with carbachol, suggesting that intercortical correlations and fronto-parietal connectivity at high gamma levels were associated with physical awakening. In addition, high gamma values of the fronto-parietal connectivity were more likely mediated by non-cholinergic mechanisms, while theta connectivity was associated with the level of cortical acetylcholine. Behavioral experiments showed that inactivated prefrontal and parietal cortices in mice could reduce the time of LORR, but only animals with an inactivated prefrontal cortex showed prolonged RR recovery time. These discrepancies highlight the frontal and parietal cortices as two discrete functional units, which roles need further studying (Pal et al., 2016; Huels et al., 2021).

#### 2.1.2 Thalamus

The centromedial nucleus of the thalamus (CMT) is a prominent population of cells of the rostral intralaminar complex that projects to most regions of the cortex, the striatum, the olfactory tubercle, and other basal nuclei. CMT may play a role in cognitive, affective, and sensorimotor functions associated with motion and non-specific arousal (Van der Werf et al., 2002; Vertes et al., 2012). Continuous sevoflurane exposure and activation of nicotinic acetylcholine receptors in the CMT led to RORR and mobility in animal models. While the application of methylamine, which is a nicotine antagonist, did not reduce the dosage of sevoflurane necessary to induce LORR, the awakening effect of nicotine was thoroughly eliminated. Furthermore, no significant arousal effects were observed after microinjecting nicotine into the paraventricular nucleus (PVN), the ventrolateral nucleus, the reticular nucleus, or the hippocampus (Alkire et al., 2007). However, experimental validation is necessary to effectively rule out these regions of the brain from being associated with consciousness modulation during sevoflurane anesthesia (Yatziv et al., 2020). In addition, the lack of observable and significant arousal effects might be due to sevoflurane anesthesia modulation through other neurotransmitters.

The Kv1 family of shaker-related (delayed rectifier) potassium channels is a sensitive molecular target of sevoflurane in CMT. Sevoflurane-anesthetized rats injected with the Kv1.2 antibody into the CMT showed partial movement recovery or RORR, while control groups did not (Alkire et al., 2009), and were successfully awakened after the injection of the ShK peptide, an inhibitor of the Kv1 family. In a heterologous expression system, sevoflurane at sub-surgical doses enhanced delayed rectifier Kv1 channel function at low depolarizing potentials and inhibited the firing rate of CMT neurons. Finally, sevoflurane delayed the onset action potential generation in thalamic mice brain slices, while the injection of ShK-186, a selective inhibitor of Kv1.3, into the CMT prevented these effects (Lioudyno et al., 2013).

These results confirm the arousal role of the CMT during sevoflurane anesthesia, in which acetylcholinergic neurons participate in the recovery process of sevoflurane anesthesia, as well as the involvement of the shaker-related potassium channel Kv1 family in the inhibition of CMT excitatory neurons.

### 2.2 Hypothalamus

#### 2.2.1 Suprachiasmatic nucleus

The suprachiasmatic nucleus (SCN) is a circadian pacemaker located in the hypothalamus of mammals. Scattered cultured single SCN neurons can present independent expression of circadian clock genes and neuronal firing (Welsh et al., 2010). The SCN projects mainly to other adjacent areas, including the periventricular projection to PVN (the subparaventricular zone of the hypothalamus, and the dorsomedial hypothalamic nucleus), and the paraventricular thalamic nuclei (PVT). Apart from regulating the circadian rhythm, the SCN-PVT-BNST (bed nucleus of the stria terminalis) circuit is associated with responses to anxiety, mood, and fear/stress (Lu and Kim, 2022). The activity of suprachiasmatic neurons can be affected by light and lead to conscious changes in sevoflurane-anesthetized mice. Specifically, exposure to monochromatic blue light (MBL) led to a significant decrease in the burst suppression ratio and a significant increase of all frequency bands in EEG except for the spindle band. Moreover, MBL significantly increased the power of local field potentials and c-Fos expression in the SCN compared to polychromatic white light (PWL), but did not alter the c-Fos expression in the locus coeruleus (LC) or the ventrolateral preoptic area (VLPO). Since the effect of MBL was removed in SCN-injured mice, the activation of SCN neurons presumably participates in accelerating sevoflurane anesthesia (Liu et al., 2020a). Similar effects were observed using acute continuous nocturnal light exposure (ACNLE). Compared to the group kept in darkness, the EEG of the ACNLE group showed a significant decrease in the frequency and duration of the burst suppression, while the peak-to-peak amplitude values of beta and gamma significantly decreased. In addition, ACNLE increased c-Fos expression and the levels of serum cortisol in SCN during sevoflurane anesthesia but did not change c-Fos expression in VLPO, suggesting SCN neurons but not VLPO activated by light stimuli are involved in light-mediated awakening following sevoflurane anesthesia (Liu et al., 2020b). The application of sevoflurane also led to a 64.5% decrease in the expression of the Per2 gene in the SCN (Ohe et al., 2011), a gene that participates in the circadian and sleep-wake rhythms through feedback regulation, and which is expressed in the peripheral nervous system and the central nervous system, including the SCN (Kim et al., 2018). Sevoflurane specifically affects the entire SCN and the expression of Per2 in a time-dependent manner through GABA signaling (Mori et al., 2014; Matsuo et al., 2016), while also decreasing histone H4 acetylation at the Per2 promoter region (Mori et al., 2014). Furthermore, bioluminescence image analysis of mPer2 promoter-destabilized luciferase transgenic rats showed that sevoflurane inhibits bioluminescence in all regions of interest and led to a phase delay. Similarly, sevoflurane-induced advancement during the rising stage but had no effect on the falling stage. In light of the above, changes in Per2 expression caused by sevoflurane and its association with consciousness require further study (Kadota et al., 2012; Anzai et al., 2013).

#### 2.2.2 Preoptic area

Despite the role of VLPO in the modulation of sevoflurane consciousness remaining unclear, the chemogenetic activation of tachykinin 1 neurons (tac1) in the preoptic area (POA) accelerates reduces the time of awakening from sevoflurane anesthesia in mice. POA is located in the anterior part of the hypothalamus and is considered a part of the sleep-wake regulation system by inhibiting nuclei from the arousal system in the TMN, the raphe nucleus, or the LC (Rothhaas and Chung, 2021). Tachykinin has been implicated in the modulation of the sleep-wake system, and intravenous tachykinin was confirmed to alter the patient’s waking time. The chemogenetic activation of POAtac1 neurons caused a rightward shift of the sevoflurane dose-response curve, while its inhibition did not increase the dose of sevoflurane needed (Reitz et al., 2021). These observations confirm that POA tac1 neurons contribute to the emergence period and promote awakening from sevoflurane anesthesia.

### 2.3 Mesolimbic system and the nucleus accumbens

#### 2.3.1 Ventral tegmental area

The ventral tegmental area (VTA) is an important component of the mesolimbic system, playing an important role in reward circuits, the formation of long-term memory, and the regulation of sleep-awake cycles through the regulation of dopaminergic pathways (Lisman and Grace, 2005; Zellner and Ranaldi, 2010). In practice, the area of mesopontine tegmental anesthesia has a strong relation with individual sensitivity to anesthetics (Minert et al., 2017), but the role of adjacent areas remains unclear. Anxiety may decrease the emergence time from sevoflurane anesthesia by affecting VTA DA neurons, as shown by a significant decrease in GCaMP6 m fluorescence values measured by Ca^2+^ signals using fiberoptic photometry in the VTA DA neurons of anxiety mice compared to controls. In addition, 90.6% of neurons expressing GCaMP6m were also positive for tyrosine dehydrogenase expression. However, the activation of VTA DA neurons by optogenetics reduced the RORR time, including in anxious mice (Wang et al., 2021), highlighting the potential effect of sevoflurane on VTA DA during anesthesia.

The rostral medial tegmental nucleus (RMTg) is the tail of the VTA and exerts a major inhibitory drive on dopamine systems (Bourdy and Barrot, 2012), modulating consciousness during sevoflurane anesthesia. The activation of RMTg neurons in mice led to decreased movements, while the mean power spectral density delta values (1–4 Hz) in EEG was similar to non-rapid oculomotor sleep periods and did not reach LORR. The concentration of sevoflurane sufficient to induce LORR decreased after the activation of RMTg neurons compared with inactivation. This remains an important subject for future research (Vlasov et al., 2021).

#### 2.3.2 Nucleus accumbens

The nucleus accumbens (NAc) mainly receives glutamatergic inputs from the prefrontal, parahippocampal, and entorhinal cortices, dopaminergic projections from the VTA, and establishes gamma-aminobutyric acid (GABA) connections with the ventral globus pallidus (Scofield et al., 2016). NAc is involved in the modulation of learning reinforcement, motivation, aversion, incentive salience, and consciousness during sevoflurane anesthesia (de Jong et al., 2022). By observing the expression of Ca^2+^, the activity of NAc neuron populations expressing the dopamine D1 receptor (D1R) started to decline before sevoflurane-induced LOC and gradually restored after ROC. After activating NAcD1R neurons using chemogenetics and optogenetics, the LORR and RORR times were prolonged and shortened, respectively, under sevoflurane exposure. At the same time, EEG showed an increase in beta and a decrease in delta. Cortical activation was also found when NAcD1R neurons were optogenetically activated. The electromyography showed awake-like behavior and muscle movement in sevoflurane-induced mice, and shifted from burst suppression to a high-frequency low-amplitude pattern (Bao et al., 2021). However, a study of amino acid expression in the NAc in rats experiencing alcohol withdrawal under different volatile anesthetics showed that sevoflurane anesthesia did not change the expression of glutamate, aspartate, and arginine in this brain region (Seidemann et al., 2017). One possible explanation is that dopamine may be involved in the modulation of consciousness during sevoflurane anesthesia in the NAc, but other excitatory neurotransmitters, such as glutamate and aspartate, are less involved in this process.

A dopaminergic pathway regulates the consciousness state in sevoflurane anesthesia from the VTA to the NAc. The extracellular DA of VTA and NAc neurons tended to rise with a decrease in Ca^2+^ activity during sevoflurane anesthesia. Retrograde tracing revealed that the NAc predominantly innervates DA neurons in the VTA. In addition, both chemogenetic and optogenetic activation showed that the activated DA pathway in VTA-NAc significantly prolonged and shortened the times to LORR and RORR, respectively, during sevoflurane anesthesia. In this process, the delta band decreased and the gamma band increased, with opposite observations in the inhibition of the VTA-NAc pathway (Gui et al., 2021). Accordingly, the activation of the VTA-NAc dopaminergic pathway could accelerate the emergence of anesthesia induced by sevoflurane.

### 2.4 Pons

#### 2.4.1 Locus coeruleus

The locus coeruleus (LC) is located in the anterior to the lateral wall of the fourth ventricle, posterolateral to the tegmental nucleus. The LC receives information from the hypothalamus, amygdala, and prefrontal cortex that provides complex emotional and cognitive information. Furthermore, the LC also receives information of visceral and sympathetic nervous system functions that are transmitted by the midbrain and brain cadres (Uematsu et al., 2015). LC noradrenergic (NE) neurons participate in the modulation of the sleep-wake cycle by receiving orexinergic projections from the lateral hypothalamus, histaminergic projections from TMN, dopaminergic projections from VTA, periaqueductal gray matter, serotoninergic projections from the dorsal raphe, cholinergic projections from the pedunculopontine tegmentum and laterodorsal tegmentum, and GABAergic projections from the VLPO and ventral lateral hypothalamus (Van Egroo et al., 2022). Sevoflurane may regulate the state of consciousness by acting on ATP-ligand-gated receptors located in the LC. A previous study showed that the application of ATP or ATPγs for 30 s in LC neuron slices of rats induced a sharp increase and moderately desensitizing inward current, whereas the use of adenosine had no such effects. These changes were significantly reduced by the application of (0.1–0.5 nm) sevoflurane in a dose-dependent manner (Masaki et al., 2001), but the drug could not continuously inhibit the inward current of LC neurons. In addition, the application of 0.5 nm sevoflurane caused early inward current in most LC neurons and significantly increased their firing rate, which was inhibited by carbenoxolone, whereby it is possible that sevoflurane leads to postoperative agitation through gap junction-related mechanisms (Yasui et al., 2007). The current changes induced by sevoflurane may occur at different time-phase, or specifically suppress the current generated by ATP-gated channels.

The LC noradrenergic system may also be involved in shaping circadian differences in sevoflurane anesthesia in mice. Specifically, minimum alveolar concentration corresponding to RORR was lower in the light phase when the mice were anesthetized with sevoflurane than in the dark phase, with a higher time of RORR. Similarly, at the rest and emergence states of sevoflurane anesthesia, the power of the delta and theta waves were higher and lower, respectively, in light vs. dark conditions. After depleting noradrenergic neurons in the LC with DSP-4, these differences disappeared (Wang et al., 2020). The differences in the modulation of consciousness in light and dark conditions were also evaluated in an article focusing on indirect projections from the SCN-DMH-LC as an arousal system in the brain (Aston-Jones et al., 2001).

#### 2.4.2 Parabrachial nucleus

There is increasing evidence that the parabrachial nucleus (PBN), an evolutionarily conserved hindbrain structure, contains a large number of glutamatergic neurons linking various threats to the accurate structures of behavioral and physiological responses. The PBN also transmits information on taste, ingestion behavior, pain, respiration, blood pressure, water balance, and thermoregulation (Chiang et al., 2019). The circuit between the PBN to the central amygdala modulates innate responses to physical stimuli and fear, while the PBN-BNST circuit regulates pain-like stress in unpredictable environments (Jaramillo et al., 2021). Chemogenetic activation of PBN glutamatergic neurons caused an increased requirement of sevoflurane in 44.4% of experimental mice animals, while their inhibition led to a leftward shift in the dose-response curve to sevoflurane in all mice. Activation of PBN glutamatergic neurons using optogenetics increased EEG frequency and decreased the amplitude rapidly. The delta and theta powers significantly decreased and increased, respectively, during the inhibition of PBN glutamatergic neurons. Moreover, the activation of PBN glutamatergic neurons also revealed increased c-Fos expression in the prefrontal cortex, lateral hypothalamus, and basal forebrain under sevoflurane anesthesia, implying that PBN glutamatergic neurons participate in the modulation of sevoflurane-induced changes in consciousness (Wang et al., 2019). PBN neurons play a regulatory function of changes in consciousness during sevoflurane anesthesia through a GABAA receptor and potassium-gated channels. After recording the medial parabrachial nucleus (MPB) calcium signal, 1.5%–3% sevoflurane decreased the MPB neuronal Ca^2+^ signal in a dose-dependent manner. During whole-cell patch-clamp recordings, the application of sevoflurane decreased the firing rate and membrane potential of MPB neurons. Finally, sevoflurane increased inhibitory postsynaptic membrane currents and directly inhibited MPB neuron activity by hyperpolarizing MPB neuron-mediated regulation of GABAA-Rs and increasing potassium conductance. In contrast, the use of PTX, a selective GABAA-R antagonist, prolonged the time of LORR and shortened the time of RORR (Xu et al., 2020), indicating that sevoflurane upregulates PBN GABAA channels and increases potassium ion conductivity.

### 2.5 Basal forebrain

#### 2.5.1 Bed nucleus of stria terminalis

The bed nucleus of stria terminalis (BNST) is composed of a bundle of axons that connects the end of the stria terminalis to the amygdala, and has extensive other connections to limbic areas of the brain, the mesolimbic system (NAc, VTA), and the prefrontal cortex. The BNST plays a key role in fear, ingestion, pain, social behavior, defensive action, and related pathophysiological processes (Dumont, 2009; Hulsman et al., 2021). A recent study showed that the PVT-BNST pathway is involved in the modulation of consciousness during sevoflurane anesthesia, with both GABAergic and glutamatergic neurons in the BNST receiving PVT glutamatergic projections. After the inhibition of the PVT-BNST pathway, the time to LORR is reduced during sevoflurane anesthesia and the dose-response curve is shifted to the left. Optogenetic activation of the PVT-BNST showed the opposite trend (Li et al., 2022).

## 3. Future Direction

Although sleep-wake is a separate process from sevoflurane anesthesia, there are numerous similarities in the modulation of brain areas (Leung et al., 2014). The regulation of anesthesia may be regulated by a number of brain areas and neurons that have been linked to sleep, and* vice versa*. A deeper knowledge of the formation of consciousness and the operation of different brain regions may result from research on the interplay between the two domains. Additionally, there is mounting proof that glial cells are involved and that sevoflurane acts on their GABA A receptors (Woll et al., 2018; Chung et al., 2022), thereby influencing their function.

It is also important to look at relationships’ deeper molecular mechanisms. Sevoflurane’s anesthetic action may be enhanced by selective D2 receptor blockade (Araki et al., 2018). Instead of directly affecting DAT, this action is believed to be mediated by inhibiting dopamine uptake (Anzawa et al., 2001), with similar effects also occurring on 5-HT (Nagatani et al., 2011). On the other hand, the existence of potent agonistic effects on arousal distinguishes orexinergic neurons from the nearby MCHergic neurons (Kelz et al., 2008). The effects of the excitatory neurotransmitter NE, however, are not as strong as those of the neurotransmitters indicated above since it exhibited no agitating conduct (Kenny et al., 2015; Pal et al., 2018). Consequently, additional research can focus on the mechanism of various receptors and neurotransmitters during sevoflurane anesthesia.

## 4. Conclusion

Both a direct and a cascade projection can be used to modulate arousal by different brain regions. Sevoflurane blocks the upward excitatory projections of the PBN and LC in the pons, the dopaminergic projections of the VTA-NAc in the midbrain, and the projections of visually associated brain areas, which blocks the basal forebrain or thalamus and ultimately the cortex. Additionally, for the express aim of providing anesthesia, sevoflurane breaks the connection between several brain areas in the cortex, such as the PFC and PC, and directly inhibits cortical activities. In summary, the thalamocortical circuit, (Pre) fronto-parietal network, CMT, SCN, POA, VTA-NAc (anterior limbic circuits), LC, PBN, and BNST, associated with a wide variety of neurotransmitters, are among the brain regions and circuits linked to alterations in consciousness under sevoflurane anesthesia (Table 1). Sevoflurane, or excruciating inhibitory output nuclei, inhibits a variety of brain arousal nuclei in a non-specific manner to suppress cortical excitability, which finally causes anesthesia. By using sevoflurane for research, we can more accurately employ it in anesthesia and comprehend the brain’s arousal circuits.

**Table 1:** Performance when brain areas is activated/inhibited during sevoflurane anesthesia.

**Brain Area**	**Main Efferent**	**Function**	**Activation**	**Inhibition**	**Neurotransmitter**	**Reference**
Prefrontal cortex	Parietal cortex; NAc; BNST	Control of body movement, Awareness; Emotion	Ach: wake like behavior; NE: EEG activation	Time to LORR↓; Time to RORR↑	Ach; NE	Alkire et al. (2008), Jäntti et al. (2008), Lee et al. (2013), Pal et al. (2017), (2018), (2020), Uhrig et al. (2018), Golkowski et al. (2019), (2021), Pappas et al. (2019), and Signorelli et al. (2021)
Parietal cortex	prefrontal cortex	/	Ach-no significant change NE: EEG activation	Time to LORR↓; No significant change in RORR	Ach; NE	Alkire et al. (2008), Jäntti et al. (2008), Lee et al. (2013), Pal et al. (2017), (2018), (2020), Uhrig et al. (2018), Golkowski et al. (2019), (2021), Pappas et al. (2019), and Signorelli et al. (2021)
CMT	Anterior and posterior regions of cortex; Claustrum; Caudate-putamen; NAc; Olfactory tubercle; Amygdala	Cognitive functions; Sensory processing; Motor functions	Righting and mobility restored	Prevent arousal response	Ach	Van der Werf et al. (2002), Alkire et al. (2007), Vertes et al. (2012), and Yatziv et al. (2020)
SCN	**(a)** a periventricular projection to PVN, SPZ, and DMH; **(b)** a rostral and posterior projection to PVT.	Circadian; Emotion; Motivation, Anxiety; Response to fear and stress	BSR↓; EEG power (especially β/γ)↑; c-Fos in PFC; LH↑	/	/	Welsh et al. (2010), Anzai et al. (2013), Liu et al. (2020a), and Lu and Kim (2022)
POA	Laterodorsal tegmental nucleus; Dorsal raphe and median raphe nucleus; LC; Ventrolateral periaqueductal gray	Coordinate sleep; Body temperature	Rightward shift of dose-response curve	no change of sensitivity of sevoflurane	Tachykinin	Reitz et al. (2021) and Rothhaas and Chung (2021)
VTA	Superior colliculus; PFC; Amygdala; Hippocampels	Primary reward	Time RORR↓; EEG:δ↓ & γ↑	/	DA	Lisman and Grace (2005), Minert et al. (2017), and Wang et al. (2021)
RMTg	/	Prediction error; Motor control; Responses to aversive stimuli and drugs of abuse	Sedation	/	GABA	Bourdy and Barrot (2012) and Vlasov et al. (2021)
NAc	Dopaminergic input from the ventral mesencephalon; Glutamatergic input from cortical, allocortical, thalamic brain regions; GABAergic projections to basal ganglia	Mediate goal-directed behaviors; Addiction	Induction time↑ Emergence time↓	Induction time↓ Emergence time↑	DA	Scofield et al. (2016), Seidemann et al. (2017), Bao et al. (2021), Gui et al. (2021), and de Jong et al. (2022)
LC	The whole brain, including the hippocampus, amygdala, thalamus, and neocortex; SCN-DMH-LC	Basic sensory; Visceral experiences; Highly processed cognitive; Emotional information	Rapidly rising and moderately desensitizing inward current induced by ATP	Changes in emergence were abolished in DSP-4-treated mice	NE	Aston-Jones et al. (2001), Masaki et al. (2001), Yasui et al. (2007), Uematsu et al. (2015), Wang et al. (2020), and Van Egroo et al. (2022)
PBN	CeA, BNST	Sensory relay; Receiving an array of interoceptive and exteroceptive inputs relevant to taste and ingestive behavior, pain, and multiple aspects of autonomic control, including respiration, blood pressure, water balance, and thermoregulation	Rightward shift of the RORR cumulative curve; Activation of cortical and subcortical arousal nuclei	Emergence time↑	Glu	Chiang et al. (2019), Wang et al. (2019), Xu et al. (2020), and Jaramillo et al. (2021)
BNST	Extensive striatal and dorsomedial prefrontal connections	Driving fear responses; Coordinating the activity of autonomic, neuroendocrine, and somatic motor systems into fully organized physiological functions and behaviors	Behavioral arousal; Reduced the depth of anesthesia burst suppression	Emergence time ↑; Induction time↓; EC50 for the RORR↓	LORR↓	Glu	Dumont (2009), Hulsman et al. (2021), and Li et al. (2022)

↑indicated that the time is increasing, the EEG power increased or the c-Fos positive cells was increased. ↓indicated that the tme is decreasing or the EEG power decreased.

## Author Contributions

GC had the idea for the article. JL and YC were in charge of researches collection. HC and GC drafted the manuscript. All authors contributed to the article and approved the submitted version.

## References

[B1] Alkire M. T. Asher C. D. Franciscus A. M. Hahn E. L. (2009). Thalamic microinfusion of antibody to a voltage-gated potassium channel restores consciousness during anesthesia. Anesthesiology 110, 766–773. 10.1097/aln.0b013e31819c461c 19322942

[B2] Alkire M. T. Hudetz A. G. Tononi G. (2008). Consciousness and anesthesia. Science 322, 876–880. 10.1126/science.1149213 18988836 PMC2743249

[B3] Alkire M. T. McReynolds J. R. Hahn E. L. Trivedi A. N. (2007). Thalamic microinjection of nicotine reverses sevoflurane-induced loss of righting reflex in the rat. Anesthesiology 107, 264–272. 10.1097/01.anes.0000270741.33766.24 17667571

[B4] Anzai M. Iijima N. Higo S. Takumi K. Matsuo I. Mori K.. (2013). Direct and specific effect of sevoflurane anesthesia on rat Per2 expression in the suprachiasmatic nucleus. PLoS One 8:e59454. 10.1371/journal.pone.0059454 23555676 PMC3605447

[B5] Anzawa N. Kushikata T. Ohkawa H. Yoshida H. Kubota T. Matsuki A. (2001). Increased noradrenaline release from rat preoptic area during and after sevoflurane and isoflurane anesthesia. Can. J. Anaesth. 48, 462–465. 10.1007/BF03028309 11394514

[B6] Araki R. Hayashi K. Sawa T. (2018). Dopamine D2-receptor antagonist droperidol deepens sevoflurane anesthesia. Anesthesiology 128, 754–763. 10.1097/ALN.0000000000002046 29251645

[B7] Aston-Jones G. Chen S. Zhu Y. Oshinsky M. L. (2001). A neural circuit for circadian regulation of arousal. Nat. Neurosci. 4, 732–738. 10.1038/89522 11426230

[B8] Bao W. W. Xu W. Pan G. J. Wang T. X. Han Y. Qu W. M.. (2021). Nucleus accumbens neurons expressing dopamine D1 receptors modulate states of consciousness in sevoflurane anesthesia. Curr. Biol. 31, 1893–1902.e5. 10.1016/j.cub.2021.02.011 33705720

[B9] Bourdy R. Barrot M. (2012). A new control center for dopaminergic systems: pulling the VTA by the tail. Trends Neurosci. 35, 681–690. 10.1016/j.tins.2012.06.007 22824232

[B10] Brown E. N. Lydic R. Schiff N. D. (2010). General anesthesia, sleep and coma. N Engl. J. Med. 363, 2638–2650. 10.1056/NEJMra0808281 21190458 PMC3162622

[B11] Brown E. N. Purdon P. L. Van Dort C. J. (2011). General anesthesia and altered states of arousal: a systems neuroscience analysis. Annu. Rev. Neurosci. 34, 601–628. 10.1146/annurev-neuro-060909-153200 21513454 PMC3390788

[B12] Chiang M. C. Bowen A. Schier L. A. Tupone D. Uddin O. Heinricher M. M. (2019). Parabrachial complex: a hub for pain and aversion. J. Neurosci. 39, 8225–8230. 10.1523/JNEUROSCI.1162-19.2019 31619491 PMC6794922

[B13] Chung W. Wang D.-S. Khodaei S. Pinguelo A. Orser B. A. (2022). GABA_A_ receptors in astrocytes are targets for commonly used intravenous and inhalational general anesthetic drugs. Front. Aging Neurosci. 13:802582. 10.3389/fnagi.2021.802582 35087395 PMC8787299

[B14] de Jong J. W. Fraser K. M. Lammel S. (2022). Mesoaccumbal dopamine heterogeneity: what do dopamine firing and release have to do with it? Ann. Rev. Neurosci. 45, 109–129. 10.1146/annurev-neuro-110920-011929 35226827 PMC9271543

[B15] Drexler B. Kreuzer M. Jordan D. Antkowiak B. Schneider G. (2013). Sevoflurane-induced loss of consciousness is paralleled by a prominent modification of neural activity during cortical down-states. Neurosci. Lett. 548, 149–154. 10.1016/j.neulet.2013.05.040 23721783

[B16] Dumont E. C. (2009). What is the bed nucleus of the stria terminalis? Prog. Neuropsychopharmacol. Biol. Psychiatry 33, 1289–1290. 10.1016/j.pnpbp.2009.07.006 19602427 PMC4011829

[B17] Franks N. P. (2008). General anaesthesia: from molecular targets to neuronal pathways of sleep and arousal. Nat. Rev. Neurosci. 9, 370–386. 10.1038/nrn2372 18425091

[B18] Gao S. Calderon D. P. (2020). Robust alternative to the righting reflex to assess arousal in rodents. Sci. Rep. 10:20280. 10.1038/s41598-020-77162-3 33219247 PMC7679463

[B19] Ghatge S. Lee J. Smith I. (2003). Sevoflurane: an ideal agent for adult day-case anesthesia? Acta Anaesthesiol. Scand. 47, 917–931. 10.1034/j.1399-6576.2003.00196.x 12904182

[B20] Golkowski D. Larroque S. K. Vanhaudenhuyse A. Plenevaux A. Boly M. Di Perri C.. (2019). Changes in whole brain dynamics and connectivity patterns during sevoflurane- and propofol-induced unconsciousness identified by functional magnetic resonance imaging. Anesthesiology 130, 898–911. 10.1097/ALN.0000000000002704 31045899

[B21] Golkowski D. Willnecker R. Rösler J. Ranft A. Schneider G. Jordan D.. (2021). Dynamic patterns of global brain communication differentiate conscious from unconscious patients after severe brain injury. Front. Syst. Neurosci. 15:625919. 10.3389/fnsys.2021.625919 34566586 PMC8458756

[B22] Gui H. Liu C. He H. Zhang J. Chen H. Zhang Y. (2021). Dopaminergic projections from the ventral tegmental area to the nucleus accumbens modulate sevoflurane anesthesia in mice. Front. Cell. Neurosci. 15:671473. 10.3389/fncel.2021.671473 33994950 PMC8119636

[B23] Guidera J. A. Taylor N. E. Lee J. T. Vlasov K. Y. Pei J. Stephen E. P.. (2017). Sevoflurane induces coherent slow-delta oscillations in rats. Front. Neural Circuits 11:36. 10.3389/fncir.2017.00036 28725184 PMC5495862

[B24] Hemmings H. C. Jr. Riegelhaupt P. M. Kelz M. B. Solt K. Eckenhoff R. G. Orser B. A.. (2019). Towards a comprehensive understanding of anesthetic mechanisms of action: a decade of discovery. Trends Pharmacol. Sci. 40, 464–481. 10.1016/j.tips.2019.05.001 31147199 PMC6830308

[B25] Hudetz A. G. (2012). General anesthesia and human brain connectivity. Brain Connect. 2, 291–302. 10.1089/brain.2012.0107 23153273 PMC3621592

[B26] Huels E. R. Groenhout T. Fields C. W. Liu T. Mashour G. A. Pal D. (2021). Inactivation of prefrontal cortex delays emergence from sevoflurane anesthesia. Front. Syst. Neurosci. 15:690717. 10.3389/fnsys.2021.690717 34305541 PMC8299111

[B27] Hulsman A. M. Terburg D. Roelofs K. Klumpers F. (2021). Roles of the bed nucleus of the stria terminalis and amygdala in fear reactions. Handb. Clin. Neurol. 179, 419–432. 10.1016/B978-0-12-819975-6.00027-3 34225979

[B28] Jäntti V. Heikkinen E. Alahuhta S. Remes R. Suominen K. (2008). Cortical electroencephalogram from subcortical electrodes rather than electrosubcorticogram. Anesthesiology 108, 963–964. 10.1097/ALN.0b013e31816bbdcf 18431134

[B29] Jaramillo A. A. Brown J. A. Winder D. G. (2021). Danger and distress: parabrachial-extended amygdala circuits. Neuropharmacology 198:108757. 10.1016/j.neuropharm.2021.108757 34461068 PMC9195487

[B30] Kadota K. Iijima N. Ohe-Hayashi Y. Takumi K. Higo S. Sakamoto A.. (2012). Time-dependent repression of mPer2 expression in the suprachiasmatic nucleus by inhalation anesthesia with sevoflurane. Neurosci. Lett. 528, 153–158. 10.1016/j.neulet.2012.07.061 22902991

[B31] Kelz M. B. Sun Y. Chen J. Cheng Meng Q. Moore J. T. Veasey S. C.. (2008). An essential role for orexins in emergence from general anesthesia. Proc. Natl. Acad. Sci. U S A 105, 1309–1314. 10.1073/pnas.0707146105 18195361 PMC2234134

[B32] Kenny J. D. Taylor N. E. Brown E. N. Solt K. (2015). Dextroamphetamine (but not atomoxetine) induces reanimation from general anesthesia: implications for the roles of dopamine and norepinephrine in active emergence. PLoS One 10:e0131914. 10.1371/journal.pone.0131914 26148114 PMC4492624

[B33] Kim M. de la Peña J. B. Cheong J. H. Kim H. J. (2018). Neurobiological functions of the period circadian clock 2 gene, *Per2*. Biomol. Ther. (Seoul) 26, 358–367. 10.4062/biomolther.2017.131 29223143 PMC6029676

[B34] Kratzer S. Mattusch C. Garcia P. S. Schmid S. Kochs E. Rammes G.. (2017). Propofol and sevoflurane differentially modulate cortical depolarization following electric stimulation of the ventrobasal thalamus. Front. Comput. Neurosci. 11:109. 10.3389/fncom.2017.00109 29321737 PMC5732174

[B35] Lee U. Ku S. Noh G. Baek S. Choi B. Mashour G. A. (2013). Disruption of frontal-parietal communication by ketamine, propofol and sevoflurane. Anesthesiology 118, 1264–1275. 10.1097/ALN.0b013e31829103f5 23695090 PMC4346246

[B36] Leung L. S. Luo T. Ma J. Herrick I. (2014). Brain areas that influence general anesthesia. Prog. Neurobiol. 122, 24–44. 10.1016/j.pneurobio.2014.08.001 25172271

[B37] Li J.-Y. Gao S.-J. Li R.-R. Wang W. Sun J. Zhang L.-Q.. (2022). A neural circuit from the paraventricular thalamus to the bed nucleus of the stria terminalis for the regulation of states of consciousness during sevoflurane anesthesia in mice. Anesthesiology 136, 709–731. 10.1097/ALN.0000000000004195 35263424

[B38] Lioudyno M. I. Birch A. M. Tanaka B. S. Sokolov Y. Goldin A. L. Chandy K. G.. (2013). Shaker-related potassium channels in the central medial nucleus of the thalamus are important molecular targets for arousal suppression by volatile general anesthetics. J. Neurosci. 33, 16310–16322. 10.1523/JNEUROSCI.0344-13.2013 24107962 PMC3792466

[B39] Lisman J. E. Grace A. A. (2005). The hippocampal-VTA loop: controlling the entry of information into long-term memory. Neuron 46, 703–713. 10.1016/j.neuron.2005.05.002 15924857

[B41] Liu D. Li J. Wu J. Dai J. Chen X. Huang Y.. (2020a). Monochromatic blue light activates suprachiasmatic nucleus neuronal activity and promotes arousal in mice under sevoflurane anesthesia. Front. Neural Circuits 14:55. 10.3389/fncir.2020.00055 32973462 PMC7461971

[B40] Liu D. Chen X. Huang Y. Zhang S. Wu J. Li J.. (2020b). Acute continuous nocturnal light exposure decreases BSR under sevoflurane anesthesia in C57BL/6J mice: possible role of differentially spared light-sensitive pathways under anesthesia. Am. J. Transl. Res. 12, 2843–2859. 32655814 PMC7344097

[B42] Lu Q. Kim J. Y. (2022). Mammalian circadian networks mediated by the suprachiasmatic nucleus. FEBS J. 289, 6589–6604. 10.1111/febs.16233 34657394

[B43] Masaki E. Kawamura M. Kato F. (2001). Reduction by sevoflurane of adenosine 5’-triphosphate-activated inward current of locus coeruleus neurons in pontine slices of rats. Brain Res. 921, 226–232. 10.1016/s0006-8993(01)03125-0 11720730

[B44] Massimini M. Ferrarelli F. Sarasso S. Tononi G. (2012). Cortical mechanisms of loss of consciousness: insight from TMS/EEG studies. Arch. Ital. Biol. 150, 44–55. 10.4449/aib.v150i2.1361 23165870

[B45] Matsuo I. Iijima N. Takumi K. Higo S. Aikawa S. Anzai M.. (2016). Characterization of sevoflurane effects on Per2 expression using *ex vivo* bioluminescence imaging of the suprachiasmatic nucleus in transgenic rats. Neurosci. Res. 107, 30–37. 10.1016/j.neures.2015.11.010 26696094

[B46] Melonakos E. D. Moody O. A. Nikolaeva K. Kato R. Nehs C. J. Solt K. (2020). Manipulating neural circuits in anesthesia research. Anesthesiology 133, 19–30. 10.1097/ALN.0000000000003279 32349073 PMC8351362

[B47] Minert A. Yatziv S.-L. Devor M. (2017). Location of the mesopontine neurons responsible for maintenance of anesthetic loss of consciousness. J. Neurosci. 37, 9320–9331. 10.1523/JNEUROSCI.0544-17.2017 28821646 PMC6596743

[B48] Mori K. Iijima N. Higo S. Aikawa S. Matsuo I. Takumi K.. (2014). Epigenetic suppression of mouse Per2 expression in the suprachiasmatic nucleus by the inhalational anesthetic, sevoflurane. PLoS One 9:e87319. 10.1371/journal.pone.0087319 24498074 PMC3909093

[B49] Nagatani H. Oshima T. Urano A. Saitoh Y. Yokota M. Nakata Y. (2011). Blockade of 5-HT(2A) and/or 5-HT(2C) receptors modulates sevoflurane-induced immobility. J. Anesthes. 25, 225–228. 10.1007/s00540-011-1103-x 21359565

[B50] Ohe Y. Iijima N. Kadota K. Sakamoto A. Ozawa H. (2011). The general anesthetic sevoflurane affects the expression of clock gene mPer2 accompanying the change of NAD+ level in the suprachiasmatic nucleus of mice. Neurosci. Lett. 490, 231–236. 10.1016/j.neulet.2010.12.059 21195744

[B51] O’Keeffe N. J. Healy T. E. (1999). The role of new anesthetic agents. Pharmacol. Ther. 84, 233–248. 10.1016/s0163-7258(99)00034-0 10665829

[B52] Pal D. Dean J. G. Liu T. Li D. Watson C. J. Hudetz A. G.. (2018). Differential role of prefrontal and parietal cortices in controlling level of consciousness. Curr. Biol. 28, 2145–2152.e5. 10.1016/j.cub.2018.05.025 29937348 PMC6039257

[B53] Pal D. Li D. Dean J. G. Brito M. A. Liu T. Fryzel A. M.. (2020). Level of consciousness is dissociable from electroencephalographic measures of cortical connectivity, slow oscillations and complexity. J. Neurosci. 40, 605–618. 10.1523/JNEUROSCI.1910-19.2019 31776211 PMC6961988

[B54] Pal D. Silverstein B. H. Lee H. Mashour G. A. (2016). Neural correlates of wakefulness, sleep and general anesthesia: an experimental study in rat. Anesthesiology 125, 929–942. 10.1097/ALN.0000000000001342 27617688 PMC5069172

[B55] Pal D. Silverstein B. H. Sharba L. Li D. Hambrecht-Wiedbusch V. S. Hudetz A. G.. (2017). Propofol, sevoflurane and ketamine induce a reversible increase in delta-gamma and theta-gamma phase-amplitude coupling in frontal cortex of rat. Front. Syst. Neurosci. 11:41. 10.3389/fnsys.2017.00041 28659769 PMC5468385

[B56] Pappas I. Cornelissen L. Menon D. K. Berde C. B. Stamatakis E. A. (2019). δ-oscillation correlates of anesthesia-induced unconsciousness in large-scale brain networks of human infants. Anesthesiology 131, 1239–1253. 10.1097/ALN.0000000000002977 31567366

[B57] Reitz S. L. Wasilczuk A. Z. Beh G. H. Proekt A. Kelz M. B. (2021). Activation of preoptic tachykinin 1 neurons promotes wakefulness over sleep and volatile anesthetic-induced unconsciousness. Curr. Biol. 31, 394–405.e4. 10.1016/j.cub.2020.10.050 33188746 PMC7855813

[B58] Rothhaas R. Chung S. (2021). Role of the preoptic area in sleep and thermoregulation. Front. Neurosci. 15:664781. 10.3389/fnins.2021.664781 34276287 PMC8280336

[B59] Sanders R. D. Tononi G. Laureys S. Sleigh J. W. (2012). Unresponsiveness ≢ unconsciousness. Anesthesiology 116, 946–959. 10.1097/ALN.0b013e318249d0a7 22314293 PMC3311716

[B60] Scofield M. D. Heinsbroek J. A. Gipson C. D. Kupchik Y. M. Spencer S. Smith A. C.. (2016). The nucleus accumbens: mechanisms of addiction across drug classes reflect the importance of glutamate homeostasis. Pharmacol. Rev. 68, 816–871. 10.1124/pr.116.012484 27363441 PMC4931870

[B61] Seidemann T. Spies C. Morgenstern R. Wernecke K. D. Netzhammer N. (2017). Influence of volatile anesthesia on the release of glutamate and other amino acids in the nucleus accumbens in a rat model of alcohol withdrawal: a pilot study. PLoS One 12:e0169017. 10.1371/journal.pone.0169017 28045949 PMC5207639

[B62] Signorelli C. M. Uhrig L. Kringelbach M. Jarraya B. Deco G. (2021). Hierarchical disruption in the cortex of anesthetized monkeys as a new signature of consciousness loss. Neuroimage 227:117618. 10.1016/j.neuroimage.2020.117618 33307225

[B63] Silva A. Amorim P. Felix L. Abelha F. Mourão J. (2018). Analysis of electroencephalogram-derived indexes for anesthetic depth monitoring in pediatric patients with intellectual disability undergoing dental surgery. J. Dent. Anesth. Pain Med. 18, 235–244. 10.17245/jdapm.2018.18.4.235 30186970 PMC6115373

[B64] Silva J. H. Gomez R. S. Diniz P. H. Gomez M. V. Guatimosim C. (2007). The effect of sevoflurane on the release of [3H]dopamine from rat brain cortical slices. Brain Res. Bull. 72, 309–314. 10.1016/j.brainresbull.2007.01.011 17452291

[B65] Tononi G. (2004). An information integration theory of consciousness. BMC Neurosci. 5:42. 10.1186/1471-2202-5-42 15522121 PMC543470

[B66] Uematsu A. Tan B. Z. Johansen J. P. (2015). Projection specificity in heterogeneous locus coeruleus cell populations: implications for learning and memory. Learn. Mem. 22, 444–451. 10.1101/lm.037283.114 26330494 PMC4561410

[B67] Uhrig L. Sitt J. D. Jacob A. Tasserie J. Barttfeld P. Dupont M.. (2018). Resting-state dynamics as a cortical signature of anesthesia in monkeys. Anesthesiology 129, 942–958. 10.1097/ALN.0000000000002336 30028727

[B68] Van der Werf Y. D. Witter M. P. Groenewegen H. J. (2002). The intralaminar and midline nuclei of the thalamus. Anatomical and functional evidence for participation in processes of arousal and awareness. Brain Res. Brain Res. Rev. 39, 107–140. 10.1016/s0165-0173(02)00181-9 12423763

[B69] Van Egroo M. Koshmanova E. Vandewalle G. Jacobs H.I.L. (2022). Importance of the locus coeruleus-norepinephrine system in sleep-wake regulation: implications for aging and Alzheimer’s disease. Sleep Med. Rev. 62:101592. 10.1016/j.smrv.2022.101592 35124476 PMC9064973

[B70] Velly L. J. Rey M. F. Bruder N. J. Gouvitsos F. A. Witjas T. Regis J. M.. (2007). Differential dynamic of action on cortical and subcortical structures of anesthetic agents during induction of anesthesia. Anesthesiology 107, 202–212. 10.1097/01.anes.0000270734.99298.b4 17667563

[B71] Vertes R. P. Hoover W. B. Rodriguez J. J. (2012). Projections of the central medial nucleus of the thalamus in the rat: node in cortical, striatal and limbic forebrain circuitry. Neuroscience 219, 120–136. 10.1016/j.neuroscience.2012.04.067 22575585

[B72] Vlasov K. Pei J. Nehs C. J. Guidera J. A. Zhang E. R. Kenny J. D.. (2021). Activation of GABAergic neurons in the rostromedial tegmental nucleus and other brainstem regions promotes sedation and facilitates sevoflurane anesthesia in mice. Anesth. Analg. 132, e50–e55. 10.1213/ANE.0000000000005387 33560660 PMC7969415

[B73] Wang D. Huang Y. Wang X. Chen X. Li J. Zhang S.. (2020). Circadian differences in emergence from volatile anaesthesia in mice: involvement of the locus coeruleus noradrenergic system. Br. J. Anaesth. 125, 548–559. 10.1016/j.bja.2020.07.012 32807382

[B75] Wang T. X. Xiong B. Xu W. Wei H. H. Qu W. M. Hong Z. Y.. (2019). Activation of parabrachial nucleus glutamatergic neurons accelerates reanimation from sevoflurane anesthesia in mice. Anesthesiology 130, 106–118. 10.1097/ALN.0000000000002475 30325744

[B74] Wang H. Yu L. Qin Y. J. Chen M. Wang X. Luo H. Q.. (2021). Restoring VTA DA neurons excitability accelerates emergence from sevoflurane general anesthesia of anxiety state. Biochem. Biophys. Res. Commun. 565, 21–28. 10.1016/j.bbrc.2021.05.079 34090206

[B76] Welsh D. K. Takahashi J. S. Kay S. A. (2010). Suprachiasmatic nucleus: cell autonomy and network properties. Ann. Rev. Physiol. 72, 551–577. 10.1146/annurev-physiol-021909-135919 20148688 PMC3758475

[B77] Woll K. A. Zhou X. Bhanu N. V. Garcia B. A. Covarrubias M. Miller K. W.. (2018). Identification of binding sites contributing to volatile anesthetic effects on GABA type A receptors. FASEB J. 32, 4172–4189. 10.1096/fj.201701347R 29505303 PMC6044061

[B78] Xu W. Wang L. Yuan X. S. Wang T. X. Li W. X. Qu W. M.. (2020). Sevoflurane depresses neurons in the medial parabrachial nucleus by potentiating postsynaptic GABA_A_ receptors and background potassium channels. Neuropharmacology 181:108249. 10.1016/j.neuropharm.2020.108249 32931816

[B79] Yasui Y. Masaki E. Kato F. (2007). Sevoflurane directly excites locus coeruleus neurons of rats. Anesthesiology 107, 992–1002. 10.1097/01.anes.0000291453.78823.f4 18043068

[B80] Yatziv S. L. Yudco O. Dickmann S. Devor M. (2020). Patterns of neural activity in the mouse brain: wakefulness vs. general anesthesia. Neurosci. Lett. 735:135212. 10.1016/j.neulet.2020.135212 32593772

[B81] Zellner M. R. Ranaldi R. (2010). How conditioned stimuli acquire the ability to activate VTA dopamine cells: a proposed neurobiological component of reward-related learning. Neurosci. Biobehav. Rev. 34, 769–780. 10.1016/j.neubiorev.2009.11.011 19914285

